# Poly (ADP) ribose polymerase enzyme inhibitor, veliparib, potentiates chemotherapy and radiation in vitro and in vivo in small cell lung cancer

**DOI:** 10.1002/cam4.317

**Published:** 2014-08-13

**Authors:** Taofeek K Owonikoko, Guojing Zhang, Xingming Deng, Michael R Rossi, Jeffrey M Switchenko, Gregory H Doho, Zhengjia Chen, Sungjin Kim, Sandy Strychor, Susan M Christner, Jan Beumer, Chunyang Li, Ping Yue, Alice Chen, Gabriel L Sica, Suresh S Ramalingam, Jeanne Kowalski, Fadlo R Khuri, Shi-Yong Sun

**Affiliations:** 1 Department of Hematology & Medical Oncology, Emory University School of MedicineAtlanta, Georgia; 2 Winship Cancer Institute of Emory UniversityAtlanta, Georgia; 3 Department of Radiation Oncology, Emory University School of MedicineAtlanta, Georgia; 4 Department of Biostatistics and Bioinformatics, Rollins School of Public Health, Emory UniversityAtlanta, Georgia; 5 Emory Integrated Genomics Core Lab, Emory University School of MedicineAtlanta, Georgia; 6 Molecular Therapeutics Drug Discovery Program, University of Pittsburgh Cancer InstitutePittsburgh, Pennsylvania; 7 Investigational Drug Branch, Cancer Therapy Evaluation Program of the National Cancer InstituteBethesda, Maryland; 8 Department of Pathology, Emory University School of MedicineAtlanta, Georgia

**Keywords:** Carboplatin, cisplatin, etoposide, PARP, SCLC, veliparib (ABT-888)

## Abstract

Poly (ADP) ribose polymerase (PARP) plays a key role in DNA repair and is highly expressed in small cell lung cancer (SCLC). We investigated the therapeutic impact of PARP inhibition in SCLC. In vitro cytotoxicity of veliparib, cisplatin, carboplatin, and etoposide singly and combined was determined by MTS in 9 SCLC cell lines (H69, H128, H146, H526, H187, H209, DMS53, DMS153, and DMS114). Subcutaneous xenografts in athymic nu/nu mice of H146 and H128 cells with relatively high and low platinum sensitivity, respectively, were employed for in vivo testing. Mechanisms of differential sensitivity of SCLC cell lines to PARP inhibition were investigated by comparing protein and gene expression profiles of the platinum sensitive and the less sensitive cell lines. Veliparib showed limited single-agent cytotoxicity but selectively potentiated (≥50% reduction in IC_50_) cisplatin, carboplatin, and etoposide in vitro in five of nine SCLC cell lines. Veliparib with cisplatin or etoposide or with both cisplatin and etoposide showed greater delay in tumor growth than chemotherapy alone in H146 but not H128 xenografts. The potentiating effect of veliparib was associated with in vitro cell line sensitivity to cisplatin (CC = 0.672; *P* = 0.048) and DNA-PKcs protein modulation. Gene expression profiling identified differential expression of a 5-gene panel (*GLS*, *UBEC2*, *HACL1*, *MSI2*, and *LOC100129585*) in cell lines with relatively greater sensitivity to platinum and veliparib combination. Veliparib potentiates standard cytotoxic agents against SCLC in a cell-specific manner. This potentiation correlates with platinum sensitivity, DNA-PKcs expression and a 5-gene expression profile.

## Introduction

Poly (ADP) ribose polymerase (PARP) is a family of enzymes that catalyze the addition of ADP-ribose to a variety of cellular constituents including DNA, histones and nonhistone proteins [1]. PARP is involved in DNA damage repair, primarily through base excision repair (BER) mechanism, important cellular machinery for repairing single strand breaks typically induced by cytotoxic therapeutic agents for small cell lung cancer (SCLC). In in vitro experiments with spontaneously immortalized mouse embryonic fibroblasts from PARP-1^(−/−)^ and PARP-1^(+/+)^ mice, PARP-1^(−/−)^-derived cells were found to be threefold more sensitive to DNA damaging agents [2]. Both genetic deletion and pharmacologic inhibition of PARP-1 sensitized cells to *γ*-radiation in vitro and in vivo suggesting a critical role of PARP-1 in the repair of radiation-induced DNA damage [3–6].

The clinical translation of PARP inhibition as a therapeutic anticancer strategy seeking to exploit the concept of synthetic lethality in genetically susceptible tumors with compromised DNA damage repair capability has not met the high degree of success anticipated [7–9]. Alternative developmental strategies of combining the PARP inhibitor class of agents with DNA damaging cytotoxic agents is now being actively pursued in the clinic [10, 11]. Veliparib (ABT-888) is a small molecule inhibitor of PARP-1 and PARP-2 enzymes and previously shown to potentiate the activity of temozolomide, cisplatin, carboplatin, and camptothecins in leukemias, gliomas, breast, and colon cancer cell lines [12–14]. This potentiating effect appears to result from concomitant increase and delayed repair of DNA damage induced by the cytotoxic agents. Veliparib and other PARP inhibitors are currently undergoing clinical evaluation in different tumor types in combination with agents such as paclitaxel/carboplatin, cisplatin/gemcitabine, topotecan, cyclophosphamide, and temozolamide [15–18].

SCLC is a lethal disease with limited treatment options [19–21]. While efforts to identify promising targeted biologic agents for the treatment of this disease continue [22], cytotoxic chemotherapy remains the mainstay of treatment. The standard frontline therapy is the combination of platinum and topoisomerase inhibitor, the cytotoxic effect of which relies largely on the induction of DNA damage leading to apoptosis [23]. In addition, SCLC has a very high level of PARP enzyme expression in comparison to other cancer types, thus suggesting a biologically relevant role for this protein in SCLC [24]. Since the optimal approach to exploit PARP enzyme inhibition as a therapeutic intervention for SCLC has not been well studied, we conducted this preclinical study to elucidate the potential therapeutic opportunities and the optimal approach to incorporating this strategy into the clinical management of SCLC.

## Materials and Methods

### Reagents

Veliparib was obtained under a material transfer agreement from the Cancer Therapy Evaluation Program (CTEP) of the National Cancer Institute and was dissolved in Dimethyl sulfoxide, aliquoted and stored at −20°F until ready for use for in vitro experiments and prepared fresh in Phosphate buffered saline (PBS) for xenograft experiments. Treatment grade samples of cisplatin, carboplatin (APP Pharmaceuticals, Schaumburg, IL), and etoposide (Teva Parenteral Medicines, Inc., Irvine, CA) were obtained from the outpatient pharmacy of the Winship Cancer Institute of Emory University. The following antibodies were employed at the indicated dilutions for Western Blot assays: actin, rabbit polyclonal, Cat# A2066, 1:3000 (Sigma-Aldrich, St. Louis, MO); ERCC1, rabbit polyclonal, cat# 3885S, 1:1000; PARP, rabbit polyclonal, Cat# 9542, 1:1000; caspase 8, mouse monoclonal, Cat# 9746, 1:1000 Ku-80, rabbit monoclonal, cat# 2180s, 1:1000; BRCA1, rabbit polyclonal, cat# 9010s, 1:1000; BRCA2, rabbit Polyclonal, cat# 9012s, 1:800; (Cell Signaling Technology, Inc., Beverly, MA); DNA-PK_cs_, mouse monoclonal, Cat# sc-5282, 1:1000; p-Histone H2A.X, rabbit polyclonal, Ser 139, Cat# sc-101696 Ku-70, mouse monoclonal, cat# sc-71470, 1:1000; GAPDH, rabbit Polyclonal, cat# sc-71470, 1:1000; (Santa Cruz Biotechnology, Dallas, TX); PAR mouse polymer monoclonal, Cat# 4335-MC-100, 1:1000 (Trevigen Inc., Gaithersburg, MD), and caspase 3, mouse monoclonal, Cat# IMG-144A, 1:500 (Imgenex Corporation, San Diego, CA).

### Cell lines and cell culture

Human SCLC cell lines (H146, H187, H128, H69, H209, DMS153, H526, DMS114, and DMS53) were purchased from the American Type Culture Collection (ATCC, Manassas, VA). Cell line purity was authenticated by short tandem repeat (STR) profiling at the Winship Cancer Institute Genomics Laboratory. The authenticity was confirmed for all cell lines except H128. The STR pattern for H128 was not consistent with the published references but consistent with the profiles for NCI-H60 (ATCC CRL-5821) and NCI-N417 (ATCC CRL-5809). It is noteworthy that we did not have the NCI-H60 and NCI-N417 in our lab throughout the duration of this work. Cells were grown as suspension or partially attached monolayer culture in RPMI 1640 medium supplemented with 5–10% fetal bovine serum at 37°C under humidified condition of 5% CO_2_ and 95% air.

### In vitro cytotoxicity

Briefly, cells were seeded in 96-well plates at ∼1–2 × 10^4^ cells per well. After 48 h, exponentially growing cells were treated by continuous exposure to vehicle, single agent or combinations of specific drugs of interest for 48–72 h. The surviving cell population following drug exposure was detected using MTS [(3-(4,5-dimethylthiazol-2-yl)-5-(3-carboxymethoxyphenyl)-2-(4-sulfophenyl)-2H-tetrazolium)/phenazine methosulfate (PMS)] colorimetric assay, Promega (Madison, WI) according to the manufacturer's protocol. Absorbance was measured at 490 nm using a microplate reader (Biotek Instruments, Winooski, VT). The 50% inhibitory concentration (IC_50_) of the cytotoxic agent in the absence and presence of veliparib was determined using GraphPad Prism software (GraphPad Software, Inc., La Jolla, CA). Combination Index (CI) analysis for drug interaction (e.g., synergy or additivity) could not be performed due to the limited activity of veliparib as a single agent in the tested cell lines. For accurate estimation of the CI using the method of Talaly and Chou, each agent in the combination is required to show single-agent activity and the dose–response curve should be sigmoidal [25]. We were unable to calculate the CI because the veliparib did not satisfy this requirement. We reported the ability of veliparib to enhance cytotoxicity of the chemotherapeutic agents and empirically defined significant potentiation of activity as ≥50% reduction in the IC_50_ concentration of a specific agent when combined with fixed concentrations of veliparib (5 and 50 *μ*mol/L).

### Western blot analysis

Preparation of whole-cell protein lysates and Western blot analysis were as previously described [26]. Expression of DNA repair pathway proteins in all the cell lines under various treatment conditions was assessed using specific antibodies targeting the protein of interest. For time course experiments, two representative cell lines with differing sensitivity to cisplatin (H146 and H128) were cultured in 100 mm^3^ plates and then exposed to vehicle, cisplatin, veliparib, and the combination of cisplatin plus veliparib. Whole-cell protein lysates prepared from cells harvested at specific time points following drug exposure (time 1, 2, 4, 12, and 24 h) were employed to assess the modulation of DNA repair proteins and markers of apoptosis. Normalized protein expression relative to actin was performed by densitometry using ImageJ, a public domain Java-based image processing software (available for download at http://imagej.nih.gov/ij/).

### In vivo tumor growth inhibition

All animal experiments were conducted in accordance with the humane treatment of animals under an animal protocol approved by the Institutional Animal Care and Use Committee (IACUC) of Emory University. Tumor xenografts were raised in 6-week-old athymic (nu/nu) mice (Harlan Industries, Indianapolis, IN) housed under pathogen-free conditions in microisolator cages, fed with laboratory chow and water ad libitum. H146 (1–2 × 10^7^) and H128 cells (2 × 10^7^) suspended in serum-free medium were mixed with matrigel solution and injected subcutaneously into the flank region of nude mice. Subcutaneously growing tumors were measured by caliper two to three times per week. When the tumors achieved a volume of ∼100 mm^3^ using the formula: [(length × width^2^)/2], groups of tumor-bearing mice (approximately six mice per group) were matched for body weight and tumor volume and randomly assigned to treatments: vehicle, veliparib (5 and 25 mg/kg o.g. daily), cisplatin (2.5 and 5 mg/kg i.p.; weekly), etoposide (20 mg/kg i.p. weekly), cisplatin plus veliparib, etoposide plus veliparib, cisplatin (2.5 mg/kg i.p. weekly) plus etoposide (20 mg/kg i.p. weekly), cisplatin (2.5 mg/kg i.p. weekly) plus etoposide (20 mg/kg i.p. weekly) plus veliparib (25 mg/kg o.g. daily). At the end of the experiments, subcutaneous tumors were harvested and weighed following animal sacrifice by cervical dislocation.

### Veliparib pharmacokinetic and platinum adducts

Tumor-bearing animals were treated with a single dose of vehicle, veliparib (5 mg/kg or 25 mg/kg), cisplatin (2.5 mg/kg or 5 mg/kg), and combinations. Treated animals were sacrificed either at 1 or 24 h posttreatment by cervical dislocation. Plasma and tumor samples were collected and immediately stored in liquid phase nitrogen or at −70°C until ready for analysis. Tissues were homogenized in approximately 1 mL of PBS. Veliparib concentrations in plasma and tissue homogenates were quantitated by LC-MS as reported previously [27]. Concentrations of total platinum in plasma and tissue homogenate were quantitated by atomic absorption spectrophotometry (AAS) as described previously [28].

### Expression profiling on Illumina HT2 and nCounter NanoString platforms and bioinformatics

Each cell line was treated with vehicle, veliparib (5 and 50 *μ*mol/L), cisplatin (determined IC_50_ concentration for each cell line), ionizing radiation (2 Gy) or cisplatin plus veliparib combination for 24 h. Total RNA was isolated from frozen specimens using RNeasy (Qiagen, Valencia, CA, USA) according to the manufacturer's instructions. Total RNA sample quality and concentrations were determined using NanoDrop and Agilent 2100 Bioanalyzer. Each sample was prepared for Illumina Human HT-12 v4 Expression BeadChips (Illumina, San Diego, CA, USA) according to the manufacturer's protocol. The HT-12 platform contains over 47,000 probes that cover well-characterized genes, gene candidates, and splice variants. BeadChips were scanned on the Illumina HiScan instrument to determine probe fluorescence intensity. Raw probe intensities for all treatment conditions were normalized by the quantile normalization algorithm [29] using GenomeStudio software from Illumina and log-2 transformed expression obtained for analyses. An unsupervised cluster analyses was done to examine the relatedness, genome-wide, among the cell lines and treatment conditions for identifying any outlying samples. Results were compared between treatment conditions to define commonly altered genes in both PARP inhibitor sensitive and insensitive cell lines.

Both a semiparametric analysis of variance (ANOVA) and a nonparametric, variance approach were implemented to obtain a robust (to analytical assumptions) gene list that was supplemented with additional genes of research interest. For the ANOVA, a mean comparison of expression was done, where feasible, to test expression differences within and among treated cell lines to controls. Results from this approach are based on an unadjusted *P* < 0.01 and a fold change of at least 1.5. Separate variance analyses were done in which empirical distributions of expression variance within each gene was performed in order to identify specific genes whose variance was among the top and bottom percentile relative to all genes (high and low variability, respectively). Genes with high expression variability among designated “sensitive” cell lines within treatment were considered as susceptible to treatment. Likewise, genes with low expression variability were considered nonresponsive to treatment. Several comparisons of results were made within and between treatments with respect to expression variability and testing for mean differences in expression based on the ANOVA results. These data were deposited in NCBI Gene Expression Omnibus as series GEO accession GSE55830.

### nCounter nanostring gene expression

The expression of 129 genes (Table S1) including 31 DNA repair genes and 38 high or low variability genes from the Illumina HT-12 expression data analysis was determined using NanoString nCounter Gene Expression platform (NanoString Technologies, Seattle WA) at the University of Miami Oncogenomics Core facility as previously described [30, 31]. The design and synthesis of probe sets for the 129 selected genes were performed at NanoString Technologies. In addition to the data from the nine cell lines, patient samples from 81 pulmonary neuroendocrine tumors (17 carcinoid, 11 large cell carcinoma, 40 small cell carcinoma, 13 neuroendocrine cancer) were included in the expression assay. Data preprocessing involved the following: an initial correction for batch assignment using the sum of the positive controls, subtraction of background signal defined by the mean expression of the negative controls, log-2 transformed, zero-centered, and quantile normalized. Samples containing greater than 75% zero expression values were removed prior to quantile normalization.

### Statistical analysis

Differences in mean IC_50_ concentrations of cytotoxic agents alone and when combined with veliparib were compared for statistical significance by ANOVA or Kruskal–Wallis test where appropriate for each cell line. Correlation between cell line sensitivity and degree of sensitivity to PARP inhibition was measured with Pearson or Spearman correlation coefficient. The effects of treatment on tumor growth rate for a given treatment relative to control group were determined as previously described using the formula %T/C = [(mean tumor volume of treated group on day X ÷ mean tumor volume of control group on day X) × 100] [12]. We assessed differences in tumor volume and rate of tumor growth overall and by pairwise comparison between different treatment groups using a mixed-effect model. Overall and pairwise differences in the harvested tumor weight across treatment groups were assessed for statistical significance by ANOVA. All analyses were performed using SAS 9.3 (SAS Institute, Inc., Cary, NC) with *P* < 0.05 considered significant.

## Results

### Veliparib displayed limited single-agent activity in vitro but potentiated the cytotoxicity of cisplatin, carboplatin, etoposide, and ionizing radiation

**Figure 1:**
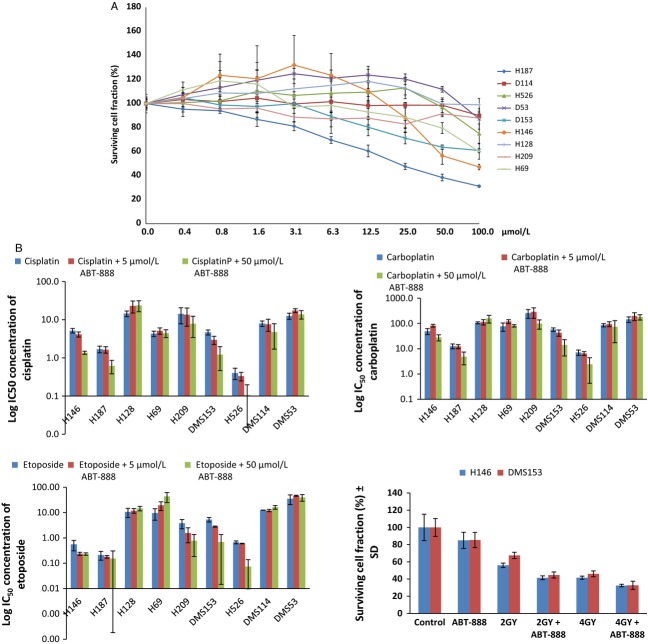
(A) Veliparib showed limited single-agent activity across a wide concentration range in a panel of SCLC cell lines. (B) Log of mean ± SEM of IC_50_ concentrations for cisplatin, carboplatin, and etoposide alone and in combination with 5 and 50 *μ*mol/L concentrations of veliparib. Each bar represents log of the mean value obtained from 3 to 4 independent experiments. Bottom Right: Potentiation of cytotoxicity induced by gamma radiation in the presence of veliparib (5 *μ*mol/L) in 2 representative cell lines (H146 and DMS153).

Short-term MTS cytotoxicity assay was performed as described in the methods section to characterize veliparib activity in a panel of 9 SCLC cell lines. We wanted to establish the single-agent activity as well as ability of veliparib to enhance the cytotoxic effect of standard chemotherapy agents employed for the treatment of SCLC patients in the clinic. Veliparib induced limited growth inhibition over a wide concentration range (0–128 *μ*mol/L) in the panel of SCLC cell lines tested (Fig. 1A). There was modest activity in several cell lines (H187, H146, DMS153) especially at concentrations ≥20 *μ*mol/L. Veliparib at a concentration of 50 *μ*mol/L but not at 5 *μ*mol/L potentiated the activity of cisplatin, carboplatin, and etoposide leading to a ≥ 50% reduction in the IC_50_ concentration of the cytotoxic drugs in five of nine cell lines (Fig. 1B and Table S2). There was a positive correlation of the magnitude of potentiation by veliparib and the sensitivity of the cell line to the cytotoxic agent, especially with cisplatin i.e., the lower the single agent IC_50_ the greater the degree of potentiation in the specific cell lines: CC = 0.67, 0.22, and 0.24 for cisplatin, carboplatin, and etoposide, respectively. Similar potentiation of radiation-induced cytotoxicity was noted when veliparib (5 *μ*mol/L) was combined with two different doses (2 and 4 Gy) of ionizing radiation in two representative cell lines (DMS153 and H146); Figure 1B.

### The combination of veliparib and cisplatin achieved greater tumor growth inhibition in SCLC xenografts

**Figure 2:**
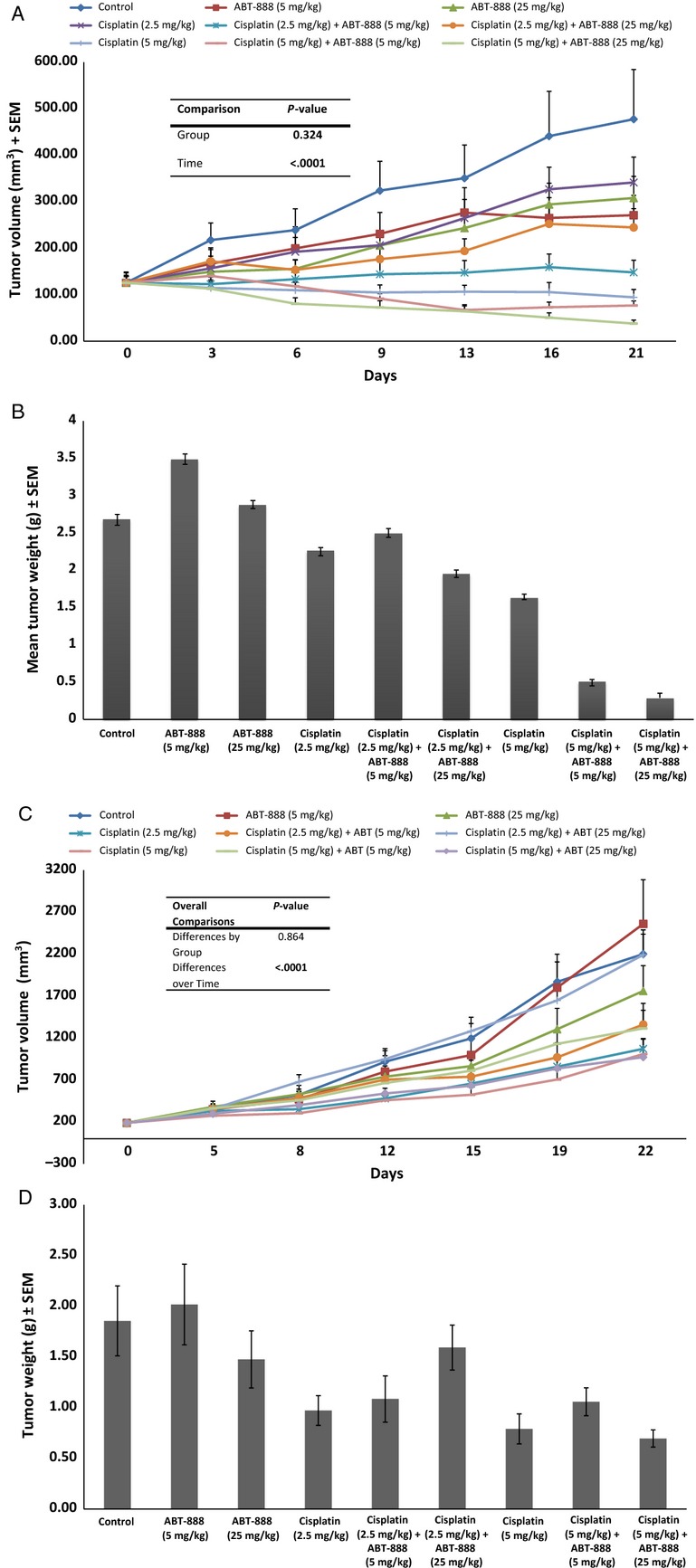
(A) H146 tumor-bearing animals were treated as indicated with vehicle, veliparib alone, cisplatin alone, and the combination of veliparib and cisplatin. Subcutaneous tumor volumes were measured at least twice weekly. The combination of veliparib with cisplatin induced greater tumor growth inhibition than cisplatin alone. (B) Animals treated with the combination of veliparib and cisplatin had the smallest tumor burden as indicated by the weights of tumor tissue harvested from euthanized mice at the end of the experiments. (C) H128 xenografts were raised in nu/nu mice. Tumor-bearing animals were treated as indicated with vehicle, veliparib alone, cisplatin alone, and the combination of veliparib and cisplatin. Subcutaneous tumor volumes were measured at least twice weekly. The combination of veliparib with cisplatin did not induce significantly greater tumor growth inhibition than cisplatin alone, similar to in vitro observations in the H128 cell line. (D) The addition of veliparib to cisplatin did not result in reduced tumor burden as indicated by the comparable weights of tumor tissue harvested from animals treated with cisplatin alone or with the combination of cisplatin and veliparib at the end of the experiments.

In order to further confirm the in vitro findings, we tested the potentiating effect of veliparib on cisplatin in vivo. We used two SCLC cell lines with a threefold difference in sensitivity to cisplatin based on the IC_50_ concentration H146 (5.2 *μ*mol/L) and H128 (14.5 *μ*mol/L) from the in vitro assay for this in vivo experiments. There was greater tumor growth inhibition with the veliparib and cisplatin combination than with cisplatin alone in H146 xenografts (Fig. 2A and B; *P* = 0.09) but not in the H128 xenograft (Fig. 2C and D; *P* > 0.1). The potentiating effect of veliparib when combined with cisplatin appeared dose dependent (Fig. 2B) but without additive toxicity as indicated by the measured weight of the animals (data not shown).

### The veliparib, etoposide, and cisplatin combination was more potent than cisplatin and etoposide alone in preventing tumor regrowth posttreatment

**Figure 3:**
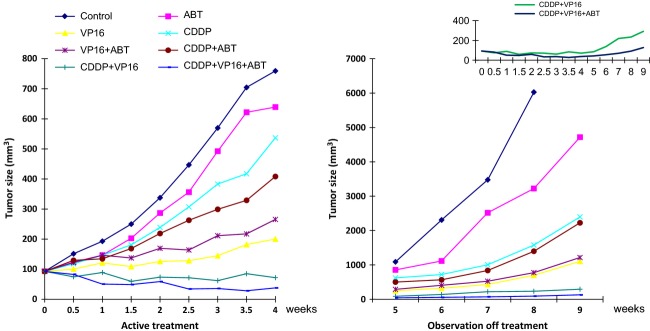
Tumor growth curves showing greater growth inhibition by doublet and triplet regimen during active treatment period (Weeks 1–4) and greater delay in tumor regrowth in animals treated with the triplet when observed off treatment (Weeks 4–9). Inset showing different tumor regrowth kinetic between doublet (cisplatin and etoposide) and triplet treatment (veliparib [25 mg/kg], cisplatin [2.5 mg/kg i.p., weekly] and etoposide [20 mg/kg i.p., weekly]); *P* < 0.021.

In order to better model the clinical management of SCLC patients where patients are typically treated with the combination of platinum and etopside and not with single-agent platinum, we wanted to test whether the addition of veliparib to the platinum doublet (cisplatin and etoposide) will result in greater antitumor effect in vivo. The triplet combination of veliparib, cisplatin, and etoposide was more potent than the doublet (*P* = 0.07) and induced objective tumor regression while the doublet only reduced tumor growth. Moreover, the triplet regimen significantly delayed tumor regrowth over the cisplatin and etoposide doublet when treated animals were observed off treatment for up to 4 weeks (*P* = 0.02; Fig. 3).

### DNA protein kinase modulation correlated with platinum sensitivity and PARP inhibitor efficacy

**Figure 4:**
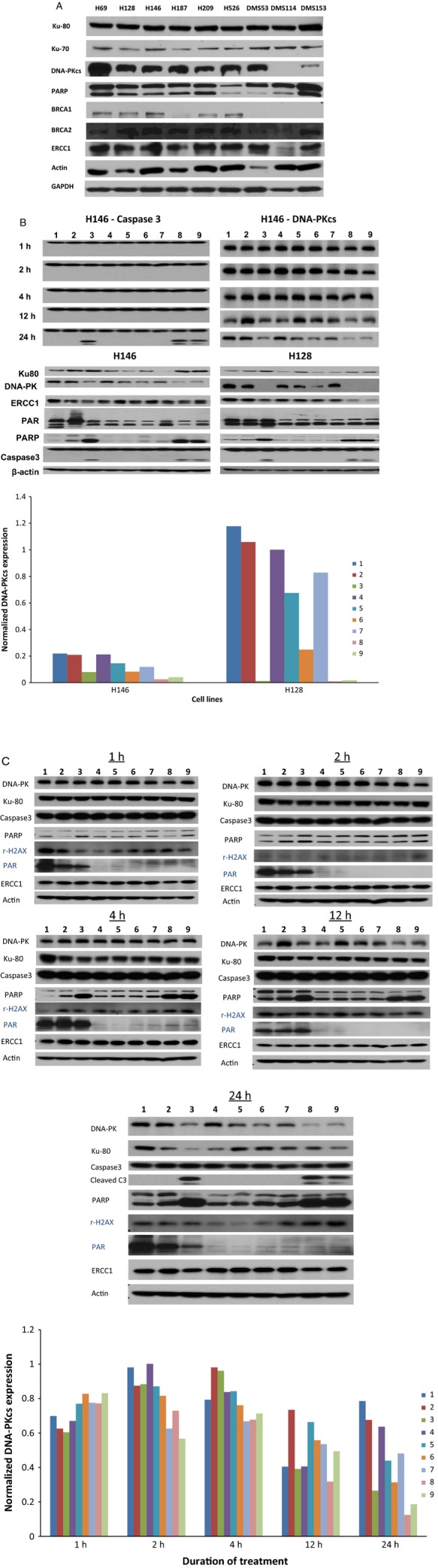
(A) Variable expression of BRCA1, ERCC1, and DNA-PKcs across the panel of SCLC cell lines. DNA-PKcs expression was noticeably high in cell lines (H69, H128 and DMS53) that were less sensitive to cisplatin or PARP inhibitor potentiation of cisplatin activity. (B) Top panel: Reduced expression of DNA-PKcs observed in H146 cell line coincided with onset of apoptosis as indicated by caspase 3 cleavage. Bottom Panel: DNA-PKcs expression was modulated in H146 and H128 cell lines but only at high concentrations of cisplatin and veliparib required for cytotoxicity (see bar graphs showing normalized DNA-PKcs expression relative to actin in H146 and H128 cell lines under different treatment conditions, 1–9, as measured by densitometry). There was no significant modulation of other representative DNA-repair enzymes. (C) Inhibition of PARP activity observed within 1 h of cell exposure to veliparib and persisted for the duration of the experiments 24 h later. Reduced DNA-PKcs expression observed at 24 h coincident with onset of apoptosis as indicated by cleaved caspase 3 and PARP (bar graphs showing normalized DNA-PKcs expression relative to actin in H146 cell lines as measured by densitometry under different treatment conditions, 1–9 and at different time points). Lanes: 1, Control; 2, cisplatin (2.5 *μ*mol/L); 3, cisplatin (50 *μ*mol/L); 4, veliparib (5 *μ*mol/L); 5, veliparib (50 *μ*mol/L); 6, cisplatin (2.5 *μ*mol/L) + veliparib (5 *μ*mol/L); 7, cisplatin (2.5 *μ*mol/L) + veliparib (50 *μ*mol/L); 8, cisplatin (50 *μ*mol/L) + veliparib (5 *μ*mol/L); 9, cisplatin (50 *μ*mol/L) + veliparib (50 *μ*mol/L).

DNA damage induced by cisplatin is mainly responsible for its cytotoxicity and anticancer effect. We expect that impaired DNA damage repair by veliparib is partly responsible for its potentiation of cisplatin cytotoxicity. We wanted to assess to whether this effect of veliparib impacts other DNA damage repair systems. We, therefore, assessed for changes in expression of DNA damage repair enzymes under different treatment conditions as described in the methods section. The native expression of BRCA1, ERCC1, and DNA-PK_cs_ was variable across the panel of cell lines (Fig. 4A). However, only DNA-PK_cs_ expression was altered when representative cell lines were treated at the optimal concentrations required for cisplatin and veliparib cytotoxicity, especially in the less sensitive H128 cell line (Fig. 4B). The down modulation of DNA-PK_cs_ coincided with the onset of apoptosis in the time course experiments using the H146 cell line as indicated by detectable caspase 3 cleavage at 24 h (Fig. 4C).

### Veliparib achieved higher intratumoral than plasma concentration in association with increased intratumoral total platinum level

**Figure 5:**
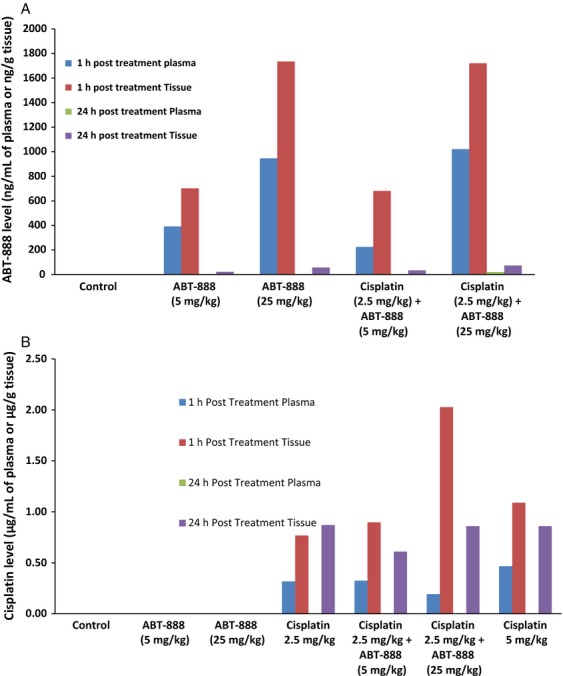
(A) Each bar represents mean levels of veliparib detected in plasma or homogenized tumor samples at 1 h and at 24 h posttreatment. Veliparib was rapidly distributed into the intratumoral compartment following oral gavage administration and was <25 ng/mL and <125 ng/g at 24 h in plasma and tissue, respectively. The pharmacokinetic property of veliparib was not significantly altered by coadministration with cisplatin. (B) Each bar represents mean levels of cisplatin detected in plasma or homogenized tumor samples at 1 h and at 24 h posttreatment in replicate mice. Note that cisplatin was no longer detectable in plasma at 24 h under all treatment conditions and that the increased intratumoral platinum observed after coadministration of cisplatin (2.5 mg/kg) and veliparib (25 mg/kg) was comparable to the level achieved with single-agent administration of cisplatin at a higher dose of 5 mg/kg.

A high concentration of veliparib of 50 *μ*mol/L was required to optimally potentiate cisplatin in our in vitro cytotoxicity assay. We wanted to determine what concentration of veliparib is achievable in vivo and whether the achievable concentration would be associated with enhanced activity of cisplatin in tumor-bearing mice. We, therefore, measured the plasma and intratumoral concentration of veliparib in tumor-bearing mice. We observed rapid clearance of veliparib from the plasma compartment following oral administration in mice. There was a twofold or higher concentration of veliparib in the tumor tissue in comparison with the plasma concentration measured at the same time point from the same animal. The exposure increased monotonically with dose although less than proportionally (Fig. 5A). Intriguingly, the intratumoral total platinum level was increased in animals treated with the combination of cisplatin and the higher dose of veliparib (25 mg/kg) when compared to cisplatin treatment alone (Fig. 5B).

### Gene expression profiling characterized SCLC cell lines sensitive to PARP inhibition

**Figure 6:**
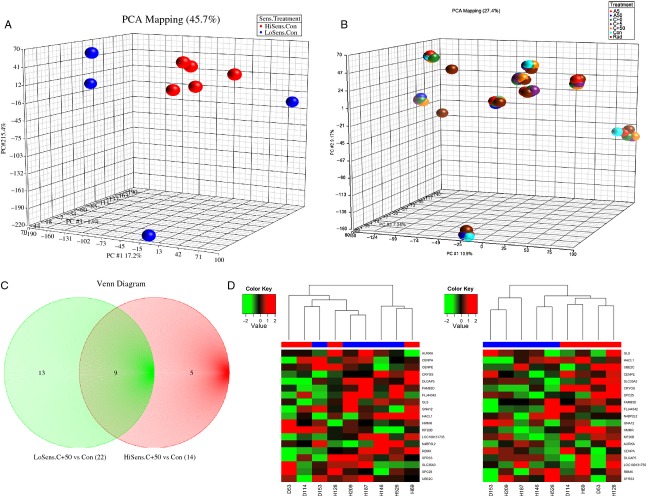
(A) Genomic DNA and RNA extracted from untreated exponentially growing cells as described were employed for gene expression profiling on the Illumina and NanoString nCounter platforms. Unsupervised analysis of the native gene expression profiles of the 9 SCLC cell lines was conducted using principal component analysis (PCA) method. Five cell lines observed to cluster together (Red) were the same cell lines that displayed increased sensitivity to cisplatin and the potentiation of cisplatin by veliparib. (B) Cells were treated by continuous exposure for 24 h to vehicle, Con, control; A5, veliparib (5 *μ*mol/L); A50, veliparib (50 *μ*mol/L); C + 0, cisplatin alone (IC_50_ concentration for the respective cell lines); C + 5, cisplatin + veliparib (5 *μ*mol/L); C + 50, cisplatin + veliparib (50 *μ*mol/L); Rad, radiation (single time point exposure to 2 Gy). Harvested cells were employed for DNA and RNA extraction as described and employed for gene expression profiling on the Illumina and NanoString nCounter platforms. PCA of the gene expression profiles of SCLC cell lines under different treatment conditions. Cells were observed to cluster principally by cell of origin rather than by specific type of treatment. (C) Venn diagram showing the distribution of the 23 differentially expressed genes and pseudo genes when untreated and treated cell lines were compared based on the sensitivity to cisplatin and PARP inhibitor potentiation of cisplatin cytotoxicity. (D) Left: Cluster analysis of Illumina expression data using the median expression value as cut off for the genes of interest showed clustering of genes according to sensitive (blue) and insensitive (red) status. Right: Cluster analysis of NanoString nCounter expression data showed separation of the cell lines into two groups, sensitive (blue) and insensitive (red).

We wanted to exploit the differential sensitivity of our panel of SCLC cell lines to PARP inhibition to gain additional insight into the biological mechanisms involved in the anticancer efficacy of this strategy. We, therefore, decided to compare the gene expression profile of the sensitive and the less sensitive cell lines in their native state and under various treatment conditions. Unsupervised cluster analysis of Illumina HT-12 data comparing the baseline gene expression profile of untreated SCLC cell lines showed tight clustering of 5 cell lines (H146, H187, H209, H526, and DMS114), which were mostly the same cell lines that displayed increased sensitivity to cisplatin and to PARP inhibition (arbitrarily defined as at least 50% reduction in the IC_50_ concentration of cisplatin when combined with veliparib; Fig. 6A). Unsupervised analysis of the gene expression profiles of the cell lines under different treatment conditions showed cells clustering by cell of origin rather than by treatment (Fig. 6B). A hierarchical supervised analysis of the gene expression profile of the two clusters of cells identified in Figure 6A (PARP inhibitor sensitive vs. PARP insensitive) before and after exposure to the optimal concentrations required for cytotoxicity i.e., cisplatin (IC_50_) and veliparib concentrations (50 *μ*mol/L), revealed a panel of 24 genes and pseudo genes (27 probe sets) with differential expression between the two cell clusters (Table S3). Five of these genes were restricted to the sensitive cell lines (*GLS*, *UBEC2*, *HACL1*, *MSI2*, and *LOC100129585*), 9 were restricted to the insensitive cell lines (*CENPE, CRYGS, FAM83D, FLJ44342, GNA12, LOC88523, LRDD, N4BP2L2, SLC35A3, SPC25*) and the remaining genes were common to both groups (*AURKA, CENPA, DLGAP5, HMMR, KIF20B, LOC100129585, LOC100131735, RBMX, SFRS3*; Fig. 6C). We speculate that this panel of genes either alone or in combination may identify the cell population likely to be sensitive to cisplatin and/or the combination of a PARP inhibitor and DNA damaging agents.

Further validation of these findings was obtained using a different gene expression platform with the nCounter NanoString technology. Unsupervised analysis of the nCounter NanoString expression data for this gene panel showed a similar clustering pattern of the cell lines as was obtained using the expression data acquired on the Illumina platform (Fig. 6D). A more robust bioinformatics analytic approach reproducibly identified a subset of this gene panel as characteristic of the platinum/PARP inhibitor sensitive and insensitive cell lines (Figs. S1 and S2).

## Discussion

This preclinical study of veliparib provides evidence in support of the strategy of targeting PARP enzyme as a potential therapy of SCLC. Emerging evidence from phospho-proteomic and genomic analysis has shown that PARP enzyme is highly expressed in SCLC and may, therefore, be a valid target for therapy [24]. Indeed, recent work evaluating BMN673, a potent PARP inhibitor, in SCLC supported the promise of PARP inhibition as a therapeutic option in SCLC [32]. Initial attempts at clinical translation of PARP inhibition relied on a strategy of synthetic lethality targeting genetically vulnerable tumors such as BRCA1- and BRCA2-deficient breast and ovarian cancers. The limitations of such an approach have become apparent due to limited efficacy of single-agent PARP inhibitor therapy [8–10, 33]. The proficient DNA damage repair capability of cancer cell lines when exposed to ionizing radiation and chemotherapeutic agents has been shown to correlate with treatment resistance [34, 35]. Given the central role of PARP enzyme in DNA damage recognition and subsequent repair by BER and its potential role in homologous recombination repair (HRR), the use of a PARP inhibitor to impede the ability of cancer cells to repair DNA damage induced by cytotoxic agents is a rational approach under intensive preclinical and clinical evaluation. We, therefore, explored whether a pharmacologic PARP inhibitor, veliparib, in combination with DNA damaging agents could potentiate therapeutic efficacy in preclinical models of SCLC.

Protein PARylation is a widely used assay to assess effective PARP enzyme inhibition [15]. We observed a significant reduction in the level of PARylated proteins in cells treated with veliparib at a concentration of 5 *μ*mol/L but optimal therapeutic potentiation when combined with DNA damaging agents required a much higher concentration of the compound. This experience is similar to prior reports showing that a higher concentration of veliparib was required for in vitro modeling of therapeutic effect [36, 37]. This then suggests either that maximal abrogation of PARP enzyme catalytic activity required to impact DNA damage occurs at a much higher concentration than required to inhibit protein PARylation or that additional cellular mechanisms beyond PARP enzyme catalytic activity are involved. Interestingly, a recently proposed mechanism for PARP inhibitor-induced cytotoxicity suggests that a tight binding of PARP to damaged DNA in cells exposed to PARP inhibitor, so called “PARP-DNA trapping,” leads to impaired DNA transcription and translation and consequent cell death [38, 39]. This mechanism appeared to be independent of the catalytic inhibitory activity and occurred at a much higher concentration of veliparib than was required for catalytic inhibition [38]. The elucidation of the possible contribution of this alternative mechanism to veliparib activity in our model systems was outside the scope of the current study. Our experimental design exposed cells to veliparib at a single time point followed by toxicity assessment 72 h later, which raises the possibility of drug degradation over time resulting in a much lower effective drug concentration at the 72-h time point. We were able to establish that veliparib was stable in cell-containing media over a 72-h period (Fig. S3) indicating that agent degradation did not impact the results of our in vitro experiments. We also determined the intratumoral veliparib concentration of 2 *μ*mol/L to be sufficient for in vivo potentiation of the antitumor effect of cisplatin, further supportive of the contention that the high in vitro concentration might be model dependent. It is worth noting that a similar model-dependent effect of PARP inhibitors was observed between short-term versus prolonged long-term in vitro assays [24]. More importantly, we observed increased intratumoral platinum concentration in the presence of veliparib, which may indicate another mechanism by which veliparib potentiates cisplatin activity. PARP inhibition was shown in experimental models of myocardial ischemia to activate the phosphoinositol-3-kinase-Akt/protein kinase B signaling pathway leading to vasodilation and increased nutrient delivery [40]. While it is plausible that increased vascular dilatation contributed to the higher levels of platinum observed in the presence of veliparib, further investigation is required to determine the relative contribution of increased platinum delivery and DNA binding, and reduced rate of DNA-platinum adduct repair to the potentiating effect of veliparib.

The selective potentiation of cytotoxic chemotherapy by veliparib in vitro and in vivo is similar to the work of other groups [36, 41]. This preclinical observation mirrors the clinical experience with SCLC, where patients manifest differential sensitivity to standard chemotherapy agents. In order to gain a clearer insight into the underlying mechanism for this differential sensitivity, we interrogated differences in other DNA repair enzymes based on the hypothesis that the activity of these complementary pathways may compensate for the consequence of PARP inhibition. We observed that DNA-PKcs expression and modulation correlated with the sensitivity of the cell lines to PARP inhibition. This observation is mechanistically relevant because DNA-PKcs is a central mediator of the nonhomologous end joining (NHEJ) DNA repair pathway, which is activated by double strand breaks resulting from the ineffective BER and HRR pathways following PARP enzyme inhibition. DNA-PKcs along with Ku70 and Ku80 form the DNA-PK holoenzyme complex, which cooperates with ATR and ATM proteins in NHEJ DNA repair. DNA damage response analysis using expression profiling identified differential ATM and ATR pathway activation in cell lines treated with veliparib in combination with topotecan in a p53-dependent manner [42]. Similarly, pharmacological inhibition of ATR using VE-821 induced greater sensitivity to veliparib in BRCA1-deficient ovarian cancer cell lines [43]. We observed that increased sensitivity to cisplatin (low IC_50_) correlates with the degree of potentiation by veliparib in our cell line panel. Clinical observation in ovarian cancer patients treated with olaparib also showed that the clinical benefit of this PARP inhibitor correlated with prior platinum sensitivity of the disease [44]. Furthermore, we observed that the relatively platinum sensitive and PARP inhibitor sensitive cell lines have lower DNA-PKcs gene and protein expression. Our data thus suggest that cells with high levels of constitutive DNA-PKcs expression might be less sensitive to DNA damaging agents. This might, however, be the result of the activity of DNA-PKcs on other cellular mechanisms in addition to DNA repair. For instance, DNA-PKcs can mediate platinum-resistance through a NHEJ pathway-independent mechanism by activating nuclear pAKT [45]. Subchronic treatment with olaparib was also shown to activate AKT in a spontaneous BRCA-deficient mouse model of breast cancer [46]. Furthermore, shRNA depletion of DNA-PKcs increased sensitivity of ovarian cancer cell lines to cisplatin while a strong association was demonstrated between cisplatin sensitivity and the expression of DNA-PKcs in patient-derived glioblastoma cell lines [47–49]. Contrarily, however, direct interaction between PARP and DNA-PKcs inhibits PARP activity while stimulating DNA-PKcs activity [6, 37, 50]. Also Patel et al. reported that an intact NHEJ repair mechanism, by generating error-prone DNA fragment and genomic instability, is necessary for the cytotoxic effect of PARP inhibition specifically in HRR-deficient cells [37]. Overall, the interaction between DNA-PKcs and PARP appears context and model dependent as evidenced by the variable effects of combined inhibition of DNA-PKcs and PARP in different model systems [51, 52]. Nonetheless, it is reasonable to anticipate that DNA-PKcs expression may offer a clinically relevant biomarker for optimal targeting of PARP inhibitors.

In conclusion, our preclinical investigation showed that PARP inhibition with veliparib potentiates the activity of DNA damaging agents in SCLC both in vitro and in vivo. The selectivity of this potentiation correlates with platinum sensitivity of the cell lines as well as the level of DNA-PKcs expression and modulation. An ongoing phase I/II clinical trial, E2511 (NCT01642251), is already translating these findings in patients with newly diagnosed SCLC. The expression profile of a 5-gene panel identified in this work may also predict both platinum sensitivity and PARP inhibitor efficacy in SCLC and potentially other tumor types, but this would have to be confirmed.

## Supporting Information

**Figure S1.** Unsupervised analysis of DNA repair gene expression from the Illumina platform. **Figure S2.** Illumina and nCounter NanoString expression data including PRKDC. **Figure S3.** Stability of ABT-888 in cell-containing media over a 72-h period.

**Table S1.** A list of 129 DNA repair and cancer-associated genes included in the ncounter nanostring gene expression assay.

**Table S2.** IC_50_ concentrations of cisplatin (*µ*mol/L), carboplatin (*µ*mol/L), and etoposide (*µ*mol/L) in the absence and presence of ABT-888.

**Table S3.** Differentially expressed genes based on the therapeutic potentiation by veliparib.
