# Supplementary material for: Poly (ADP) ribose polymerase enzyme inhibitor, veliparib, potentiates chemotherapy and radiation in vitro and in vivo in small cell lung cancer
Source: Cancer Med. 2014 Aug 13;3(6):1579–94. doi: 10.1002/cam4.317 (PMC4298385; doi:10.1002/cam4.317)
Supplement: Supplementary file 1 — Figure S1. Unsupervised analysis of DNA repair gene expression from the Illumina platform. Figure S2. Illumina and nCounter NanoString expression data including PRKDC. Figure S3. Stability of ABT-888 in cell-containing media over a 72-h period. [file cam40003-1579-sd1.pptx]

## Slide 1
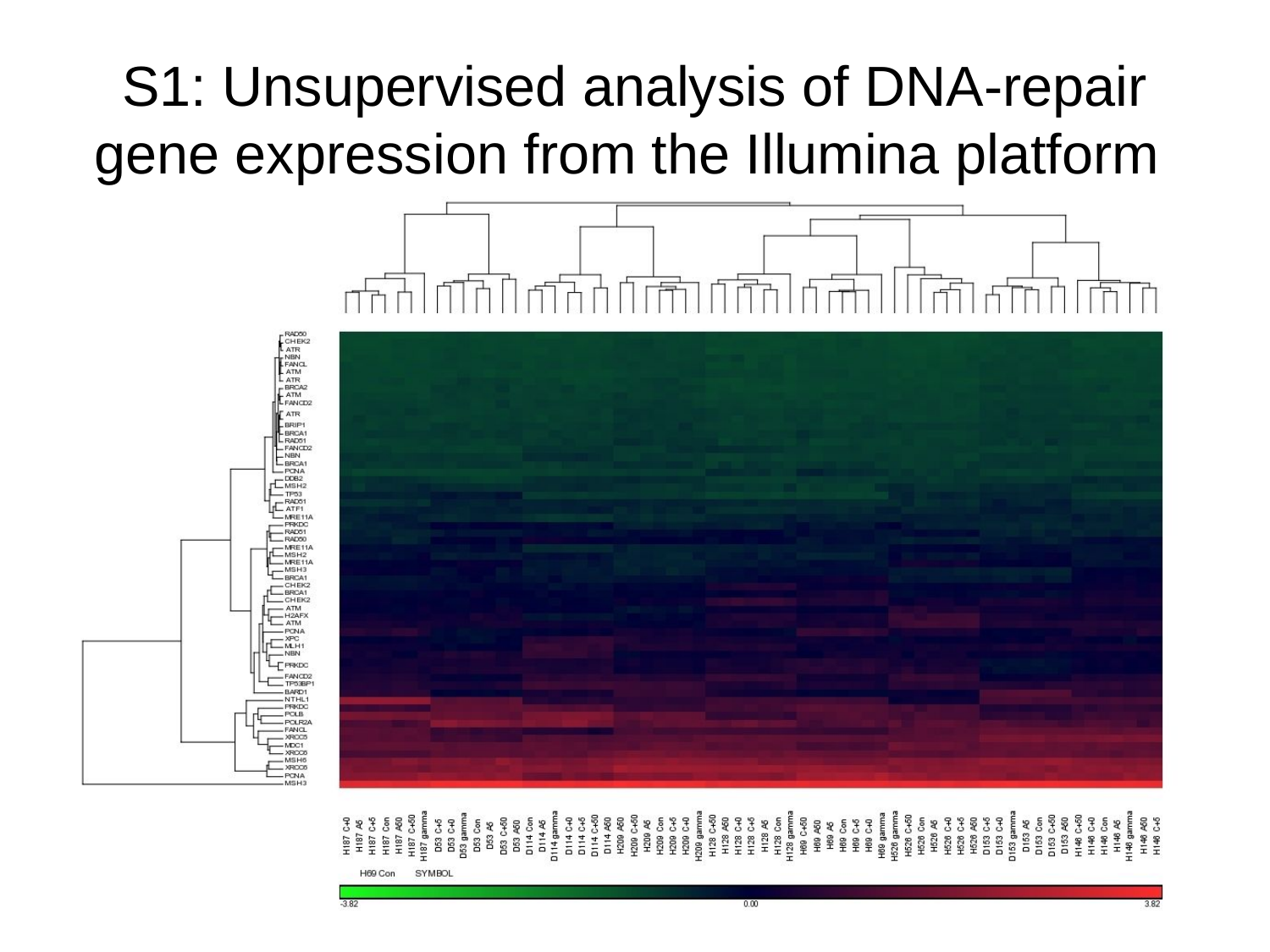

# S1: Unsupervised analysis of DNA-repair gene expression from the Illumina platform

## Slide 2
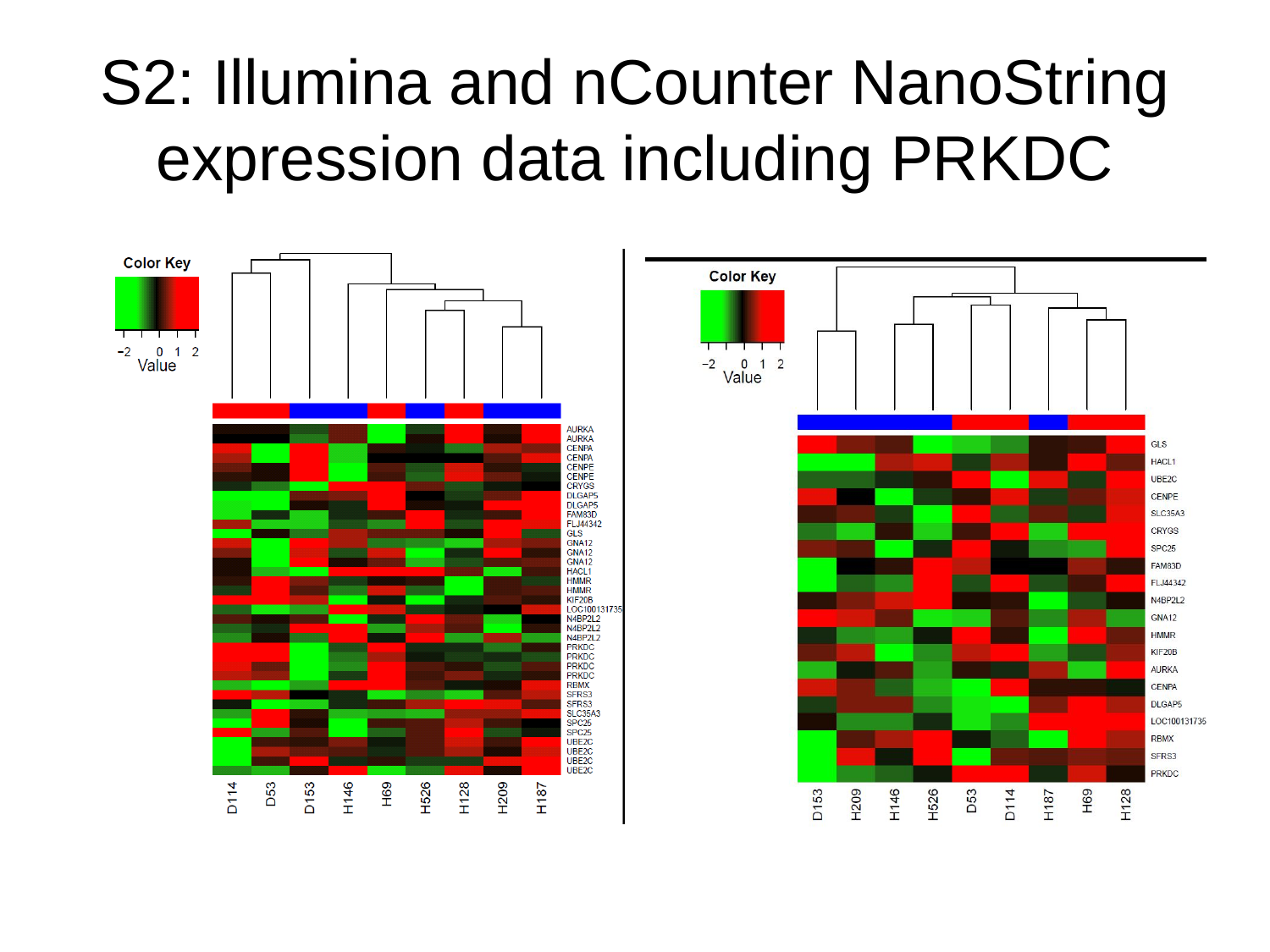

# S2: Illumina and nCounter NanoString expression data including PRKDC

## Slide 3
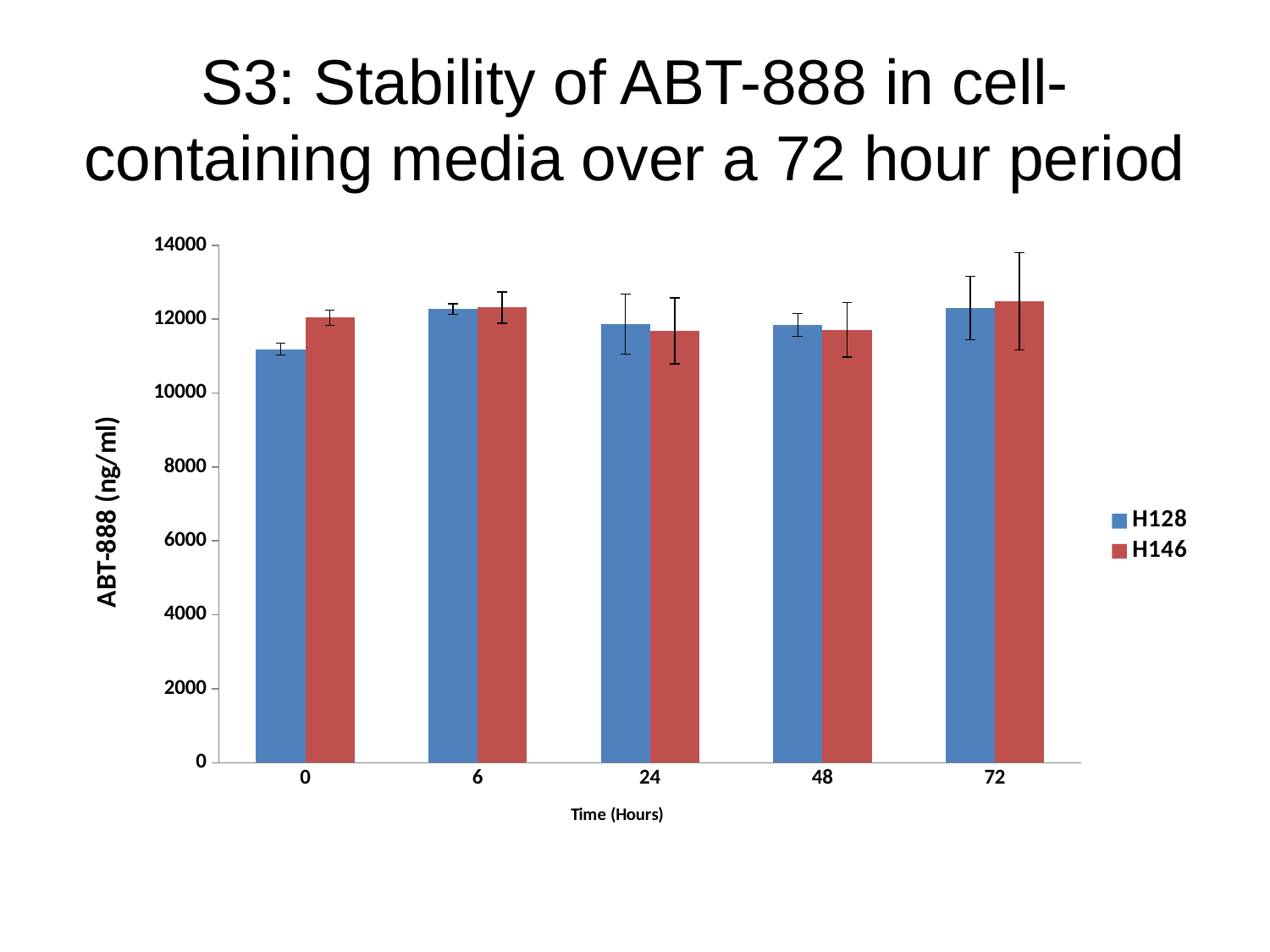

# S3: Stability of ABT-888 in cell-containing media over a 72 hour period
### Chart
| Category | H128 | H146 |
|---|---|---|
| 0 | 11191.209103712727 | 12039.10513395496 |
| 6 | 12271.871575003488 | 12312.972978964548 |
| 24 | 11868.450240470573 | 11686.327274643927 |
| 48 | 11847.562409314953 | 11717.018557617814 |
| 72 | 12303.65290930676 | 12485.369587318439 |
