# Supplementary material for: Poly (ADP) ribose polymerase enzyme inhibitor, veliparib, potentiates chemotherapy and radiation in vitro and in vivo in small cell lung cancer
Source: Cancer Med. 2014 Aug 13;3(6):1579–94. doi: 10.1002/cam4.317 (PMC4298385; doi:10.1002/cam4.317)
Supplement: Supplementary file 2 — Table S1. A list of 129 DNA repair and cancer-associated genes included in the ncounter nanostring gene expression assay. [file cam40003-1579-sd2.doc]

**Table S1: A list of 129 DNA-Repair and Cancer Associated Genes Included in the nCounter NanoString Gene Expression Assay**

| ***Gene*** | ***Probe NSID*** | ***Tot Isoforms*** | ***Isoforms Hit by Probe*** |
| --- | --- | --- | --- |
| ALCAM | NM_001627.3:789 | 4 | NM_001627;NM_001243283;NM_001243281;NM_001243280 |
| ATF1 | NM_005171.2:710 | 1 | NM_005171 |
| ATM | NM_138292.3:6688 | 1 | NM_138292;NM_000051 |
| ATP2B4 | NM_001684.3:7640 | 2 | NM_001684;NM_001001396 |
| ATR | NM_001184.2:565 | 1 | NM_001184 |
| AURKA | NM_003600.2:405 | 6 | NM_003600;NM_198437;NM_198436;NM_198435;NM_198434;NM_198433 |
| AZIN1 | NM_148174.2:505 | 2 | NM_148174;NM_015878 |
| BARD1 | NM_000465.2:302 | 1 | NM_000465 |
| BEX5 | NM_001159560.1:196 | 2 | NM_001159560;NM_001012978 |
| BLM | NM_000057.2:2135 | 1 | NM_000057 |
| BLOC1S1 | NM_001487.3:382 | 4 | NM_001487;NR_037656;NR_037655;NR_037657 |
| BRCA1 | NM_007305.2:1275 | 6 | NM_007305;NR_027676;NM_007299;NM_007298;NM_007297;NM_007300;NM_007294 |
| BRCA2 | NM_000059.3:115 | 1 | NM_000059 |
| BRIP1 | NM_032043.1:1130 | 1 | NM_032043 |
| C2orf42 | NM_017880.1:465 | 1 | NM_017880 |
| C5orf28 | NM_022483.4:146 | 1 | NM_022483 |
| CASP3 | NM_032991.2:685 | 2 | NM_032991;NM_004346 |
| CBY1 | NM_001002880.1:920 | 2 | NM_001002880;NM_015373 |
| CCBL2 | NM_001008661.2:1445 | 2 | NM_001008661;NM_001008662 |
| CCNB2 | NM_004701.3:333 | 1 | NM_004701;XR_012150;CD766722;XM_510447 |
| CD3EAP | NM_012099.1:555 | 1 | NM_012099 |
| CENPA | NM_001042426.1:979 | 2 | NM_001042426;NM_001809 |
| CENPE | NM_001813.2:291 | 1 | NM_001813 |
| CHEK2 | NM_007194.3:140 | 3 | NM_007194;NM_001005735;NM_145862 |
| CLU | NM_001831.2:2340 | 3 | NM_001831;NR_038335;NM_203339;NR_045494 |
| CMIP | NM_198390.2:710 | 2 | NM_198390;NM_030629 |
| COL11A2 | NM_001163771.1:760 | 4 | NM_001163771;NM_080681;NM_080679;NM_080680 |
| COX17 | NM_005694.1:5 | 1 | NM_005694 |
| CRYGS | NM_017541.2:115 | 1 | NM_017541 |
| DCLRE1B | NM_022836.3:1260 | 1 | NM_022836 |
| DDB2 | NM_000107.1:840 | 1 | NM_000107 |
| DDX39A | NM_005804.2:1287 | 2 | NM_005804;NR_038336 |
| DLEU2 | NR_002612.1:1394 | 1 | NR_002612 |
| DLGAP5 | NM_014750.3:130 | 2 | NM_014750;NM_001146015 |
| DSCR6 | NM_018962.2:395 | 1 | NM_018962 |
| E2F1 | NM_005225.1:935 | 1 | NM_005225 |
| ERCC1 | NM_001983.3:458 | 3 | NM_001983;NM_001166049;NM_202001 |
| ERCC2 | NM_000400.2:240 | 2 | NM_000400;NM_001130867 |
| ERCC3 | NM_000122.1:1950 | 1 | NM_000122 |
| ERCC4 | NM_005236.2:1700 | 1 | NM_005236 |
| ERCC5 | NM_000123.2:1630 | 1 | NM_000123 |
| ERCC6 | NM_000124.2:3235 | 1 | NM_000124 |
| ERCC8 | NM_000082.3:175 | 1 | NM_000082 |
| EXO1 | NM_003686.3:2715 | 3 | NM_003686;NM_006027;NM_130398 |
| EYA3 | NM_001990.2:675 | 1 | NM_001990 |
| FAM72B | NM_001100910.1:283 | 1 | NM_001100910 |
| FAM83D | NM_030919.2:865 | 1 | NM_030919 |
| FANCD2 | NM_033084.3:260 | 2 | NM_033084;NM_001018115 |
| FANCL | NM_001114636.1:446 | 2 | NM_001114636;NM_018062 |
| FGF12 | NM_004113.4:685 | 2 | NM_004113;NM_021032 |
| FLJ44342 | XR_109412.1:4780 | 1 | XR_109412 |
| GATS | NM_178831.6:1220 | 4 | NM_178831;NR_028040;NR_028039 |
| GLS | NM_014905.3:985 | 1 | NM_014905;NM_001256310 |
| GNA12 | NM_007353.2:4180 | 1 | NM_007353 |
| H2AFX | NM_002105.2:1392 | 1 | NM_002105 |
| HACL1 | NM_012260.2:1325 | 1 | NM_012260 |
| HDAC3 | NM_003883.2:1455 | 1 | NM_003883 |
| HMMR | NM_012484.2:100 | 4 | NM_012484;NM_001142557;NM_001142556;NM_012485 |
| HNRNPH3 | NM_021644.3:1065 | 2 | NM_021644;NM_012207 |
| KDM4C | NM_015061.2:2665 | 4 | NM_015061;NM_001146696;NM_001146695;NM_001146694 |
| KIF20B | NM_016195.2:1885 | 1 | NM_016195 |
| LARP1B | NM_178043.1:305 | 3 | NM_178043;NM_032239;NM_018078 |
| LOC100131735 | XR_133514.1:16 | 1 | XR_133514 |
| MCOLN3 | NM_001253693.1:176 | 1 | NM_001253693;NM_018298 |
| MDC1 | NM_014641.2:6719 | 1 | NM_014641 |
| MFSD3 | NM_138431.1:1335 | 1 | NM_138431 |
| MGMT | NM_002412.3:323 | 1 | NM_002412 |
| MICALCL | NM_032867.2:2250 | 1 | NM_032867 |
| MLH1 | NM_000249.2:1605 | 4 | NM_000249;NM_001167619;NM_001167618;NM_001167617 |
| MRE11A | NM_005591.3:505 | 2 | NM_005591;NM_005590 |
| MSH2 | NM_000251.1:2105 | 1 | NM_000251 |
| MSH3 | NM_002439.2:2555 | 1 | NM_002439 |
| MSH6 | NM_000179.1:3525 | 1 | NM_000179 |
| MSI2 | NM_138962.2:347 | 2 | NM_138962;NM_170721 |
| MTSS1 | NM_014751.4:2725 | 1 | NM_014751 |
| MUS81 | NM_025128.4:2116 | 1 | NM_025128 |
| MYT1 | NM_004535.2:3240 | 1 | NM_004535 |
| MZT1 | NM_001071775.2:365 | 1 | NM_001071775 |
| N4BP2L2 | NM_014887.2:1558 | 2 | NM_014887;NM_033111 |
| NBN | NM_001024688.1:1105 | 1 | NM_001024688;NM_002485 |
| NDFIP2 | NM_019080.2:400 | 2 | NM_019080;NM_001161407 |
| NMNAT3 | NM_178177.2:1260 | 2 | NM_178177;NM_001200047 |
| NTHL1 | NM_002528.5:476 | 1 | NM_002528 |
| OVOL2 | NM_021220.2:676 | 1 | NM_021220 |
| PARP1 | NM_001618.3:3016 | 1 | NM_001618 |
| PCNA | NM_002592.2:280 | 2 | NM_002592;NM_182649 |
| PDDC1 | NM_182612.2:1235 | 1 | NM_182612 |
| PIDD | NM_145886.2:2133 | 2 | NM_145886;NM_145887 |
| POLB | NM_002690.1:145 | 1 | NM_002690 |
| POLR2A | NM_000937.2:3775 | 1 | NM_000937 |
| POLR2B | NM_000938.1:1835 | 1 | NM_000938 |
| POLR2E | NM_002695.3:1450 | 1 | NM_002695 |
| PRDM4 | NM_012406.3:875 | 1 | NM_012406 |
| PRKDC | NM_006904.6:12750 | 2 | NM_006904;NM_001081640 |
| RAC1 | NM_198829.1:1250 | 2 | NM_198829;NM_018890;NM_006908 |
| RAD50 | NM_005732.2:5397 | 1 | NM_005732 |
| RAD51 | NM_133487.2:566 | 4 | NM_133487;NM_001164270;NM_001164269;NM_002875 |
| RASSF6 | NM_177532.3:1985 | 2 | NM_177532;NM_201431 |
| RBBP8 | NM_002894.2:760 | 3 | NM_002894;NM_203292;NM_203291 |
| RBMX | NM_002139.3:137 | 4 | NM_002139;NR_028477;NR_028476;NM_001164803 |
| RHBDD2 | NM_001040456.1:274 | 2 | NM_001040456;NM_001040457 |
| RNF165 | NM_152470.2:1450 | 1 | NM_152470;NM_001256758 |
| RPA1 | NM_002945.3:950 | 1 | NM_002945 |
| RPA2 | NM_002946.3:505 | 1 | NM_002946 |
| S100A13 | NM_001024210.1:567 | 5 | NM_001024210;NM_001024213;NM_001024212;NM_001024211;NM_005979 |
| SART3 | NM_014706.3:1195 | 1 | NM_014706 |
| SRSF3 | NM_003017.4:2640 | 2 | NM_003017;NR_036610 |
| SRSF7 | NM_001031684.2:532 | 2 | NM_001031684;NM_001195446 |
| SGPP2 | NM_152386.2:850 | 1 | NM_152386 |
| SIX4 | NM_017420.3:6030 | 1 | NM_017420 |
| SLC22A23 | NM_021945.5:5185 | 2 | NM_021945;NM_015482 |
| SLC35A3 | NM_012243.1:605 | 1 | NM_012243 |
| ANKRD43 | NM_175873.4:1990 | 1 | NM_175873 |
| SPC25 | NM_020675.3:326 | 1 | NM_020675 |
| STK35 | NM_080836.2:2075 | 1 | NM_080836 |
| TERF1 | NM_003218.3:1037 | 2 | NM_003218;NM_017489 |
| TGFBRAP1 | NM_001142621.1:1585 | 2 | NM_001142621;NM_004257 |
| TMCO3 | NM_017905.4:2070 | 1 | NM_017905 |
| TMEM30B | NM_001017970.2:2420 | 1 | NM_001017970 |
| TP53 | NM_000546.2:1330 | 7 | NM_000546;NM_001126117;NM_001126116;NM_001126115;NM_001126114;NM_001126113;NM_001126112 |
| TP53BP1 | NM_005657.2:132 | 3 | NM_005657;NM_001141980;NM_001141979 |
| TPD52 | NM_001025252.1:710 | 3 | NM_001025252;NM_005079;NM_001025253 |
| UBE2C | NM_181803.1:269 | 6 | NM_181803;NM_007019;NM_181802;NM_181800;NM_181799;NM_181801 |
| UBL3 | NM_007106.2:35 | 1 | NM_007106 |
| USMG5 | NM_032747.3:392 | 3 | NM_032747;NM_001206427;NM_001206426 |
| XPC | NM_004628.3:2135 | 3 | NM_004628;NR_027299;NM_001145769 |
| XRCC5 | NM_021141.3:832 | 1 | NM_021141 |
| XRCC6 | NM_001469.3:670 | 1 | NM_001469 |
| XRCC6BP1 | NM_033276.2:445 | 1 | NM_033276 |
