# Supplementary material for: Poly (ADP) ribose polymerase enzyme inhibitor, veliparib, potentiates chemotherapy and radiation in vitro and in vivo in small cell lung cancer
Source: Cancer Med. 2014 Aug 13;3(6):1579–94. doi: 10.1002/cam4.317 (PMC4298385; doi:10.1002/cam4.317)
Supplement: Supplementary file 3 — Table S2. IC50 concentrations of cisplatin (µmol/L), carboplatin (µmol/L), and etoposide (µmol/L) in the absence and presence of ABT-888. [file cam40003-1579-sd3.doc]

**Table S2: IC_50_ concentrations of cisplatin (µM), carboplatin (µM) and etoposide (µM) in the absence and presence of ABT-888**

| ***Cell line*** | ***Cisplatin*** | ***Cisplatin**** | ***Cisplatin#*** | ***Carboplatin*** | ***Carboplatin**** | ***Carboplatin#*** | ***Etoposide*** | ***Etoposide**** | ***Etoposide#*** |
| --- | --- | --- | --- | --- | --- | --- | --- | --- | --- |
| H146^ | 5.2±0.7 | 4.1±0.6 | 1.4±0.1 | 49.6±13.1 | 82.6±10.3 | 27.9±7.6 | 0.6±0.3 | 0.2±0.03 | 0.2±0.03 |
| H187^ | 1.7±0.3 | 1.6±0.3 | 0.6±0.2 | 12.7±2.7 | 12.4±2.3 | 4.9±2.5 | 0.2±0.1 | 0.2±0.02 | 0.2±0.2 |
| H128 | 14.5±2.4 | 23.0±8 | 23.7±7.6 | 106.4±9.6 | 115.4±27.8 | 157.4±53.3 | 10.6±4.2 | 11.9±2.4 | 14.6±3 |
| H69 | 4.3±0.7 | 5.1±1 | 4.4±1.0 | 76.9±27.8 | 120.8±21.4 | 81.2±8.8 | 9.6±4.6 | 19.4±7.4 | 43.5±18.7 |
| H209^ | 14.2±6.4 | 13.5±6.8 | 7.9±4.5 | 258.1±99.8 | 288.6±128.5 | 99.2±39.7 | 3.8±1.5 | 1.6±0.9 | 0.8±0.6 |
| DMS153^ | 4.7±0.7 | 3.0±0.7 | 1.2±0.8 | 57.8±9.2 | 43.2±11.9 | 14.1±8.9 | 5.3±1.1 | 2.8±0.1 | 0.7±0.7 |
| H526^ | 0.4±0.1 | 0.3±0.1 | 0.1±0.1 | 7.2±1.7 | 6.6±1.2 | 2.4±2.0 | 0.7±0.1 | 0.6±0.01 | 0.07±0.06 |
| DMS114 | 8.0±1.3 | 7.6±2.7 | 4.8±3.1 | 85.8±12.8 | 96.2±21.4 | 73.7±56.3 | 12.5±0.1 | 12.1±1.3 | 16.3±2.7 |
| DMS53 | 12.5±2.3 | 17.4±2.1 | 13.8±3.5 | 146.2±36.4 | 198.0±67.5 | 179.3±43.3 | 35.3±14.7 | 46.4±2.1 | 39.7±11.5 |
| - - combined with veliparib (5µM) - # - combined with veliparib (50µM) - ^ - significant therapeutic potentiation (>50% reduction in IC50 of chemotherapy agent in the presence of ABT-888) observed. | | | | | | | | | |
