# Supplementary material for: Poly (ADP) ribose polymerase enzyme inhibitor, veliparib, potentiates chemotherapy and radiation in vitro and in vivo in small cell lung cancer
Source: Cancer Med. 2014 Aug 13;3(6):1579–94. doi: 10.1002/cam4.317 (PMC4298385; doi:10.1002/cam4.317)
Supplement: Supplementary file 4 — Table S3. Differentially expressed genes based on the therapeutic potentiation by veliparib. [file cam40003-1579-sd4.doc]

**Table S3: Differentially expressed genes based on therapeutic potentiation by veliparib**

| *SYMBOL* | *p-value (Treatment vs. Control)* | *Ratio (Treatment vs. Control)* | | *Fold-Change (Treatment vs. Control)* |
| --- | --- | --- | --- | --- |
| Genes with Significant Change in Expression Profile within the PARP inhibitor Insensitive Cell Lines | | | | |
| LOC100129585 | 0.000323 | | 0.581426 | -1.71991 |
| RBMX | 0.000447 | | 0.589658 | -1.6959 |
| GNA12 | 0.002389 | | 0.666187 | -1.50108 |
| DLGAP5 | 0.001412 | | 0.502178 | -1.99133 |
| LOC100131735 | 0.000421 | | 0.58341 | -1.71406 |
| FLJ44342 | 0.000127 | | 1.55278 | 1.55278 |
| CENPA | 0.002023 | | 0.521589 | -1.91722 |
| HMMR | 0.000304 | | 0.610075 | -1.63914 |
| DLGAP5 | 0.005133 | | 0.568203 | -1.75993 |
| KIF20B | 0.002885 | | 0.66522 | -1.50326 |
| CRYGS | 0.000113 | | 1.56508 | 1.56508 |
| FAM83D | 0.006619 | | 0.629408 | -1.58879 |
| SPC25 | 0.004633 | | 0.65665 | -1.52288 |
| SFRS3 | 0.04031 | | 0.650251 | -1.53787 |
| AURKA | 0.012884 | | 0.617344 | -1.61984 |
| LOC88523 | 0.029342 | | 2.13811 | 2.13811 |
| N4BP2L2 | 0.030554 | | 1.77569 | 1.77569 |
| AURKA | 0.012674 | | 0.597865 | -1.67262 |
| CENPE | 0.02366 | | 0.656467 | -1.52331 |
| LRDD | 0.044125 | | 1.55844 | 1.55844 |
| CENPE | 0.043524 | | 0.623035 | -1.60504 |
| SLC35A3 | 0.028143 | | 0.664018 | -1.50598 |
|  |  | |  |  |
| Genes with Significant Change in Expression Profile within the PARP inhibitor Sensitive Cell Lines | | | | |
| SYMBOL | p-value (Treatment vs. Control) | Ratio (Treatment vs. Control) | | Fold-Change (Treatment vs. Control) |
| LOC100129585 | 4.75E-07 | 0.627205 | | -1.59437 |
| LOC100129585 | 1.50E-05 | 0.547442 | | -1.82668 |
| RBMX | 6.68E-05 | 0.578664 | | -1.72812 |
| DLGAP5 | 0.000266 | 0.488912 | | -2.04536 |
| LOC100131735 | 0.000163 | 0.594342 | | -1.68253 |
| CENPA | 0.001669 | 0.552114 | | -1.81122 |
| UBE2C | 0.00156 | 0.610259 | | -1.63865 |
| DLGAP5 | 0.005688 | 0.607084 | | -1.64722 |
| SFRS3 | 0.011304 | 0.618099 | | -1.61786 |
| GLS | 0.017325 | 1.6567 | | 1.6567 |
| AURKA | 0.009993 | 0.638952 | | -1.56506 |
| AURKA | 0.02376 | 0.66041 | | -1.51421 |
| HACL1 | 0.007434 | 0.640178 | | -1.56207 |
| MSI2 | 0.030249 | 0.578437 | | -1.7288 |
